# 

**Published:** 1912-07

**Authors:** 


### INDEX TO VOLUME XXVIII.

A New Therapeutic Agent in the Treatment of Pulmonary Tuberculosis. By Sevier Warren, M. D., M. R. C., San Angelo, Texas .................................... 505
Alcohol. By William Francis Waugh, A. M., M. D......... 184
Abstracts and Selections......34, 80, 120, 213, 258, 306, 347, 444, 483 ........................................ 531
Books and Magazines....37, 86, 130, 218, 264, 310, 350, 398, 491
Can the Increase of Insanity be Prevented? By Wilmer L. Allison, M. D., Fort Worth, Texas....................... 5
Chronic Gastritis. By W. T. Marrs, M. D., Peoria, Ill....... 48
Communications ..................................32, 392
Department of Eugenics......21, 52, 97, 146, 187, 236, 278, 319, 372, 416, 459.................................... 510
Editorial Department.......27, 73, 113, 167, 209, 252, 299, 340, 389, 434, 479.................................... 525
Editorial Notes, News and Miscellany......29, 76, 117, 168, 213, 256, 303, 344, 391, 442, 480...................... 531
General Eye Remedies. By W. T. Marrs, M. D........... 454
Hookworm Disease or Uncinariasis—Its Symptoms, Prevalence and Proposed Methods of Eradication. By M. H. Boerner, M. D., Commissioner in Charge............... 106
How Can We Hold Our Husbands? or Suggestions for the Correction of the "Divorce Evil." By a Wife and Mother.... 227
Medical Fraternalism and Medical Citizenship. By Alexander S. Garrett, M. D., Springtown, Texas.................. 1
Obstructed Nasal Breathing in Infancy and Childhood—Is There Developing Among Us a Modern Race of Mouth Breathers? By E. Mather Sill, M. D., New York City.... 45
Observations in the Course of the Life of an Active M. D. By A. F. Sampson, M. D., San Francisco, Cal.............. 405
Observations on Inguinal Hernia. By L. Sexton, B. S., M. D., New Orleans, La................................ 359
On the Restoration of the Alamo. By Daniel Parker, M. D.. 455
Proserpine ("Mrs. Pluto") on the Divorce Evil........... 269
Publisher's Department......38, 86, 130, 176, 222, 265, 312,
Publisher's Department......38, 86, 130, 176, 222, 265, 312, 356, 400, 496........................................ 542
Radical Reform, A ..................................... 340

431958

Reminiscences of Hawaii—En Route for Captain Cook's Monument. By John C. Warbrick, M. D., Chicago............ 451
Rheumatism and Tuberculosis. By R. H. L. Bibb, M. D., Austin, Texas.................................... 315
Should Sex Hygiene be Taught in the Home and School? By Frederick Eby, Austin, Texas...................... 97
Some Handicaps Which the Country Doctor May Overcome. By B. G. R. Williams, M. D., Paris, Ill.................. 139
Some Interesting and Probably Original Surgical Procedures. By A. F. Sampson, M. D., San Francisco, Cal........... 89
Sterilizing the Unfit. By G. Henri Bogart, M. D., Paris, Ill.. 370
Surgical Solution of the Problem of Race Culture. By Malone Duggan, M. D., San Antonio, Texas.................... 499
Tetanus. By G. B. Taylor, M. D........................ 459
The Kernel of the Nut, or an Answer to "Proserpine." By A Wife and Mother.................................. 366
The Prevention of the Increase of Insanity, of the Procreation of the Congenitally Defective and of the Criminally Disposed. By Daniel Parker, M. D., Calvert, Texas.......... 135
The Venereal Problem—A Quintette of Laws............. 181
Typhoid Vaccine in the Treatment of Typhoid Fever—Report of Cases. By Marvin B. Grace, M. D., Seguin, Texas...... 12
Woman's Department..17, 63, 106, 159, 195, 245, 289, 333, 381, 425, 471....................................... 519

### The Woman's Department.

**CONDUCTED BY MRS. F. E. DANIEL**

ASSISTED BY

MRS. ISABELLA CHARLES DAVIS

of New Jersey

DR. MARGARET HOLLIDAY
Women's Physician, University of Texas

MRS. JNO. S. TURNER

And others whose names will be announced later

### VITAL FACTS EVERY WOMAN SHOULD KNOW.

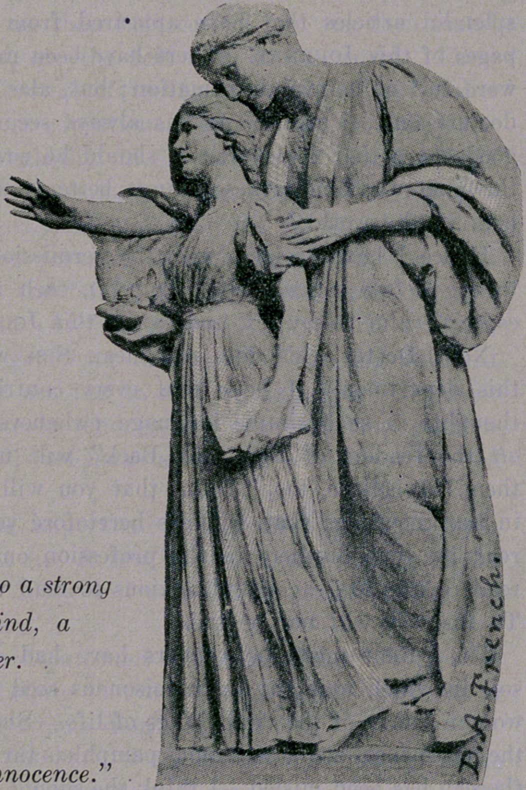

*Teach me the way to a strong body, a pure mind, a noble character.*

*"Ignorance is not innocence."*

